# Suppression of allograft rejection by CD8+CD122+PD-1+ Tregs is dictated by their Fas ligand-initiated killing of effector T cells *versus* Fas-mediated own apoptosis

**DOI:** 10.18632/oncotarget.15551

**Published:** 2017-02-20

**Authors:** Huazhen Liu, Yeshu Wang, Qiaohuang Zeng, Yu-Qun Zeng, Chun-Ling Liang, Feifei Qiu, Hong Nie, Zhenhua Dai

**Affiliations:** ^1^ Section of Immunology, Guangdong Provincial Academy of Chinese Medical Sciences, and Guangdong Provincial Hospital of Chinese Medicine, Guangzhou, Guangdong, P.R. China; ^2^ Graduate School, Guangzhou University of Chinese Medicine, Guangzhou, Guangdong, P.R. China; ^3^ Student Exchange Program, Mayo Clinic, Rochester, MN, USA; ^4^ Guangdong Provincial Key Laboratory of Pharmacodynamic Constituents of TCM and New Drugs Research, College of Pharmacy, Jinan University, Guangzhou, Guangdong, P.R. China

**Keywords:** transplantation, Treg, immunoregulation, T cell apoptosis, Immunology and Microbiology Section, Immune response, Immunity

## Abstract

Mounting evidence has shown that naturally occurring CD8+CD122+ T cells are regulatory T cells (Tregs) that suppress both autoimmunity and alloimmunity. We have previously shown that CD8+CD122+PD-1+ Tregs not only suppress allograft rejection, but also are more potent in suppression than conventional CD4+CD25+ Tregs. However, the mechanisms underlying their suppression of alloimmunity are not well understood. In an adoptive T-cell transfer model of mice lacking lymphocytes, we found that suppression of skin allograft rejection by CD8+CD122+PD-1+ Tregs was mostly dependent on their expression of Fas ligand as either lacking Fas ligand or blocking it with antibodies largely abolished their suppression of allograft rejection mediated by transferred T cells. Their suppression was also mostly reversed when effector T cells lacked Fas receptor. Indeed, these FasL+ Tregs induced T cell apoptosis *in vitro* in a Fas/FasL-dependent manner. However, their suppression of T cell proliferation *in vitro* was dependent on IL-10, but not FasL expression. Furthermore, adoptive transfer of CD8+CD122+PD-1+ Tregs significantly extended allograft survival even in wild-type mice if Tregs lacked Fas receptor or if recipients received recombinant IL-15, as these two measures synergistically expanded adoptively-transferred Tregs in recipients. Thus, this study may have important implications for Treg therapies in clinical transplantation.

## INTRODUCTION

CD4+CD25+ regulatory T cells (Treg) prevent allograft rejection and are essential for tolerance in animal models [1–8]. However, mounting evidence has demonstrated that naturally occurring CD8+CD122+ T cells are also Tregs that inhibit conventional T cell responses [9–14], antitumor immunity [15], as well as autoimmunity [16, 17]. We have previously shown that CD8+CD122+ T cells are not only Tregs [18, 19], but also are more potent in suppression of allograft rejection than are conventional CD4+CD25+ Tregs [20]. In particular, we have demonstrated that PD-1-positive component within CD8+CD122+ T cell population is mainly responsible for their regulatory activities while antigen-specific CD8+CD122+PD-1- T cells are memory T cells [18]. Therefore, CD8+CD122+ Tregs likely correspond to their CD4+CD25+ counterparts since CD122 is the β subunit of IL-2 receptor on T cells while CD25 is the α subunit of the same receptor [21]. More accurately, CD8+CD122+PD-1+ Tregs likely correspond to their CD4+CD25+FoxP3+ counterparts, and they probably cooperate to maintain the immunologic homeostasis and keep autoimmune responses in check. However, the mechanisms underlying the suppression of alloimmunity by CD8+CD122+PD-1+ Tregs remain not well understood, although it has been shown that IL-10 is partially involved in their suppression of allograft rejection [18]. Therefore, it is imperative to fully understand the mechanisms responsible for the Treg suppression so that they can be fully exploited to inhibit allograft rejection in an immune competent recipient or even in humans.

In an adoptive T-cell transfer model of Rag1−/− mice, we found that suppression of skin allograft rejection in by CD8+CD122+PD-1+ Tregs was mostly dependent on their expression of Fas ligand. Their suppression was also largely reversed when effector T cells lacked Fas receptor. The FasL+ Tregs indeed induced conventional T cell apoptosis *in vitro* in a FasL-Fas-dependent manner. Moreover, the Treg adoptive transfer extended allograft survival even in wild-type mice when the Tregs themselves lacked Fas receptor or if recipients received recombinant IL-15 since these two approaches synergistically expanded Tregs that were transferred to wild-type recipients.

## RESULTS

### Fas ligand expression on CD8+CD122+PD-1+ Tregs is critical for their suppression of allograft rejection

**Figure 1:**
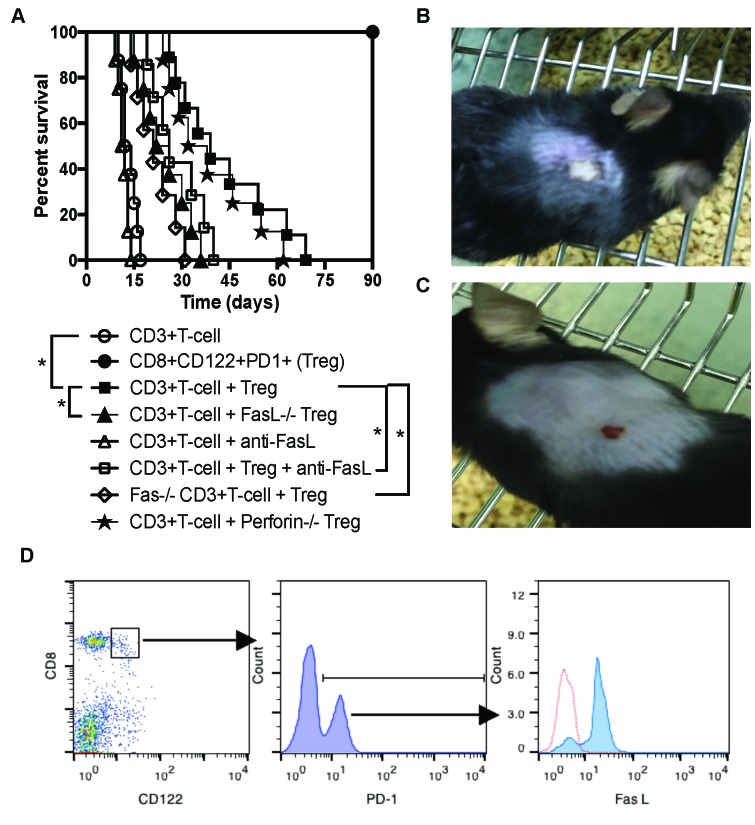
FasL/Fas pathway plays an important role for CD8+CD122+PD-1+ Treg-mediated suppression of skin allograft rejection (**A**.) Shown is skin allograft survival after various treatments. Rag1−/− mice (B6 background) were transplanted with a piece of Balb/C skin and received syngeneic CD3+ T cells (*n* = 8), CD8+CD122+PD-1+ Tregs (*n* = 8) or both (*n* = 9) with a Treg/Teff ratio of 1:4. Some recipients received the Tregs derived from FasL−/− (*n* = 8) or Perforin−/− mice (*n* = 8) while others received T cells as effectors from Fas−/− (lpr) mice (*n* = 7). Additional recipients were also treated with a blocking anti-FasL antibody (*n* = 7). (*P < 0.05, *n* = 7-9). (**B**. & **C**.) One representative of accepted or rejected skin allograft is shown. (**D**.) CD8+CD122+PD-1+ Tregs isolated from naïve B6 mice mostly expressed FasL prior to their adoptive transfer, as determined by flow analyses. One of two separate flow data is shown.

To search for the mechanisms underlying immunosuppression mediated by memory-like CD8+CD122+PD-1+ Tregs, we determined a role for Fas ligand (FasL) in their suppression of allograft rejection. Rag1−/− mice on B6 background were transplanted with a Balb/C skin graft and received syngeneic CD3+ T cells and/or CD8+CD122+PD-1+ Tregs. Some recipients received the Tregs derived from FasL−/− (gld) mice while others were treated with a blocking anti-FasL antibody. As shown in Figure 1A, the transfer of CD8+CD122+PD-1+ Tregs significantly delayed skin allograft rejection mediated by CD3+ T cells (MST= 39 vs. 13 days, n=8-9, P<0.05). As controls, transfer of the Tregs alone did not reject the allografts. However, the suppression of allograft rejection by CD8+CD122+PD-1+ Tregs was mostly diminished by either utilization of FasL-deficient Tregs (MST= 24 vs. 39 days, n=8, P<0.05) or treatments with a blocking anti-FasL mAb (MST= 26 vs. 39 days, n=7-8, P<0.05). Isotype control mAb did not alter the allograft survival (data not shown). Moreover, the Tregs were much less effective in suppression of allograft rejection when CD3+ effector T cells lacked Fas receptor (MST= 21 vs. 39 days, n=7-8, P<0.05). On the other hand, a lack of perforin on the Tregs did not alter their capacity to prolong skin allograft survival. Shown also was a representative image of accepted (Figure 1B) or rejected (Figure 1C) skin allograft. Indeed, most of the purified CD8+CD122+PD-1+ Tregs expressed FasL prior to their adoptive transfer (Figure 1D). Thus, these data indicate that FasL/Fas, but not perforin/granzyme, pathway plays an important role in CD8+CD122+PD-1+ Treg-mediated suppression of allograft rejection.

### CD8+CD122+PD-1+ Tregs promote CD3+ effector T cell apoptosis in a FAS/FasL-dependent manner

**Figure 2:**
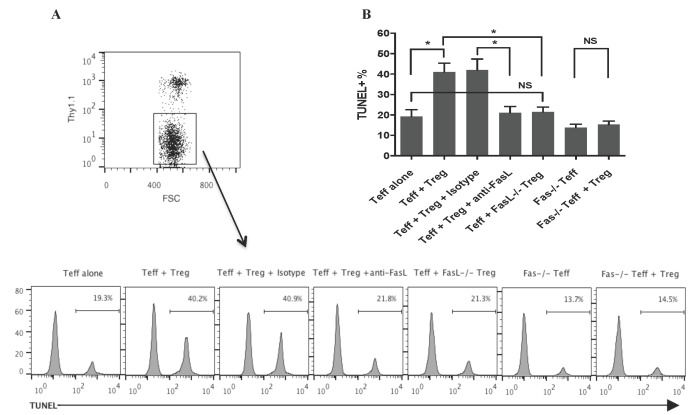
CD8+CD122+PD-1+ Tregs induce CD3+ T cell apoptosis ***in vitro*** in a FAS/FasL-dependent manner. FACS-sorted CD8+CD122+PD-1+ Tregs derived from Thy1.1+ mice and CD3+ Thy1.1- T cells (Teff) were cultured and activated by anti-CD3 and anti-CD28 Abs for 72 hours. The ratios of Treg to Teff were 1:4. Some cultures were also treated with a blocking anti-FasL antibody. Thy1.1-negative Teff cells were then analyzed for their apoptosis using a TUNEL method, as described in the methods. Histograms shown are gated on a Thy1.1- population from one representative of three TUNEL experiments (**A**.) Bar graphs represent the percentage of apoptotic cells (mean ± SD) pooled from three independent experiments (**B**.) (*P < 0.05, NS denotes non-significant).

Since we found that Fas-FasL pathway was critical for CD8+CD122+PD-1+ Treg-mediated suppression of allograft rejection, we asked whether or not CD8+CD122+PD-1+ Tregs would directly induce effector T cell apoptosis via engagement of Fas-FasL pathway. FACS-sorted CD8+CD122+PD-1+Thy1.1+ Tregs and CD3+ Thy1.1- T cells were cultured and activated by anti-CD3 and anti-CD28 mAbs for 72 hours. Thy1.1- T cells were then analyzed for their apoptosis using a TUNEL method. As shown in Figure 2A & 2B, CD8+CD122+PD-1+ Tregs significantly induced effector T cell apoptosis while their FasL-deficient counterparts failed to do so. Similarly, anti-FasL blocking mAb reversed T cell apoptosis induced by the Tregs when compared to the isotype control. On the other hand, CD8+CD122+PD-1+ Tregs also failed to promote the apoptosis of Fas-deficient T cells. These findings suggest that CD8+CD122+PD-1+ Tregs induce the apoptosis of effector T cells via interactions between their surface FasL and the Fas receptor on effector T cells.

### Fas/FasL pathway is not required for CD8+CD122+PD-1+ Treg suppression of T cell proliferation

**Figure 3:**
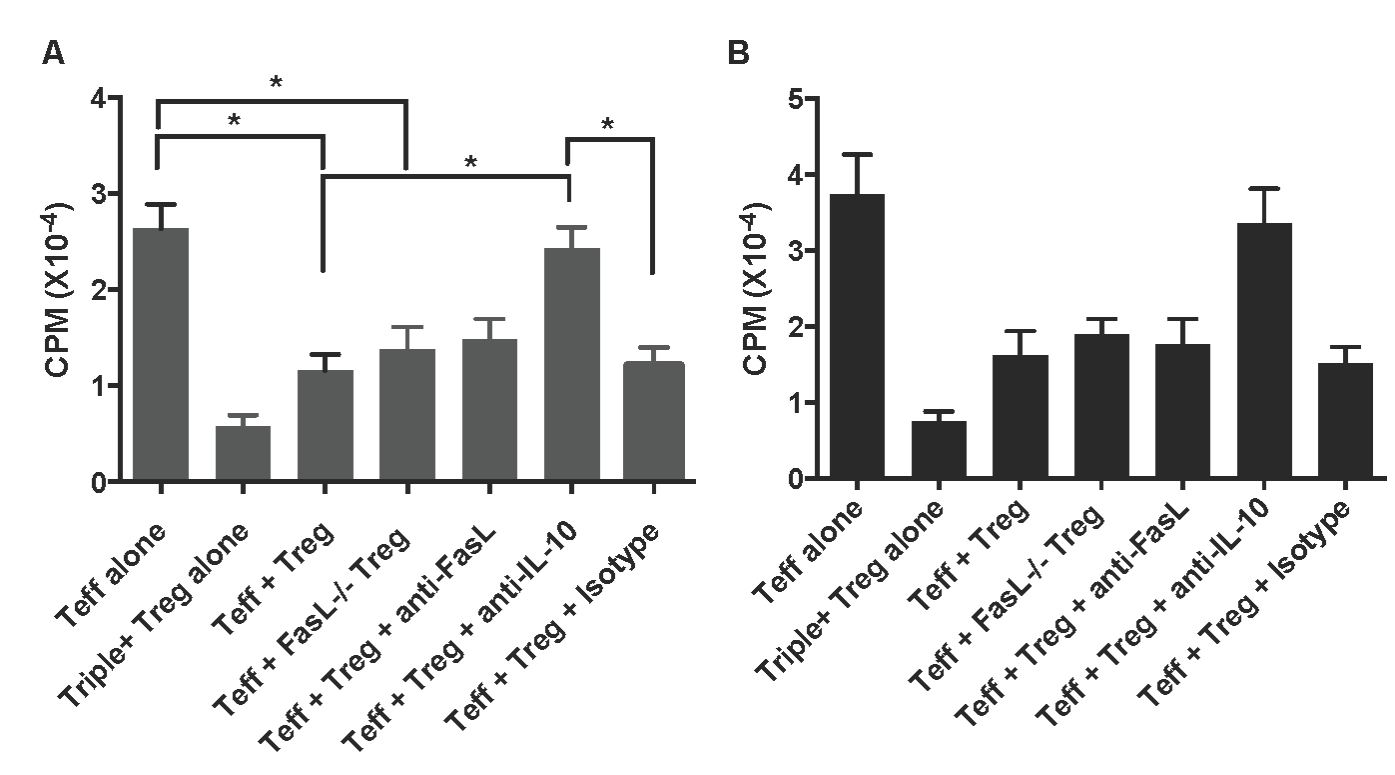
CD8+CD122+PD-1+ Treg suppression of T cell proliferation ***in vitro***. One-way MLR was set up using CD8+CD122+PD-1+ Tregs as suppressors, enriched T cells as responders or effectors (Teff), and irradiated Balb/C splenocytes as stimulators. The ratios of Treg to Teff were 1:4. In some groups, cell cultures were treated with anti-FasL or anti-IL-10 mAb, as described in the methods. T cell proliferation was analyzed using a thymidine-uptake method three (**A**.) and five (**B**.) days after the culture. Data are presented as mean ± SD. One representative of two separate experiments is shown.

To determine whether or not Fas signaling also plays a role in suppression of T cell proliferation *in vitro* by CD8+CD122+PD-1+ Tregs, one-way MLR was set up using these Tregs as suppressors, enriched T cells as responders or effectors (Teff), and irradiated Balb/C splenocytes as stimulators. In some groups, cell cultures were treated with anti-FasL or anti-IL-10 mAb. As shown in Figure 3A, CD8+CD122+PD-1+ (Triple+) Tregs drastically inhibited T cell (Teff) proliferation three days following the culture. Interestingly, neither lack of FasL on the Tregs nor anti-FasL blocking mAb significantly altered the Treg suppression of T cell proliferation, indicating that Fas/FasL signaling pathway is not required for CD8+CD122+PD-1+ Treg-mediated suppression of T cell proliferation. However, neutralizing IL-10 abolished their inhibition of T cell proliferation, suggesting that IL-10, but not Fas/FasL interaction, is essential for the Treg-mediated suppression of T cell proliferation. The same findings were seen five days following the cell culture (Figure 3B).

### Depriving Tregs of Fas death signaling or supplying recipients with recombinant IL-15 inhibits allograft rejection in immunologically competent wild-type mice

**Figure 4:**
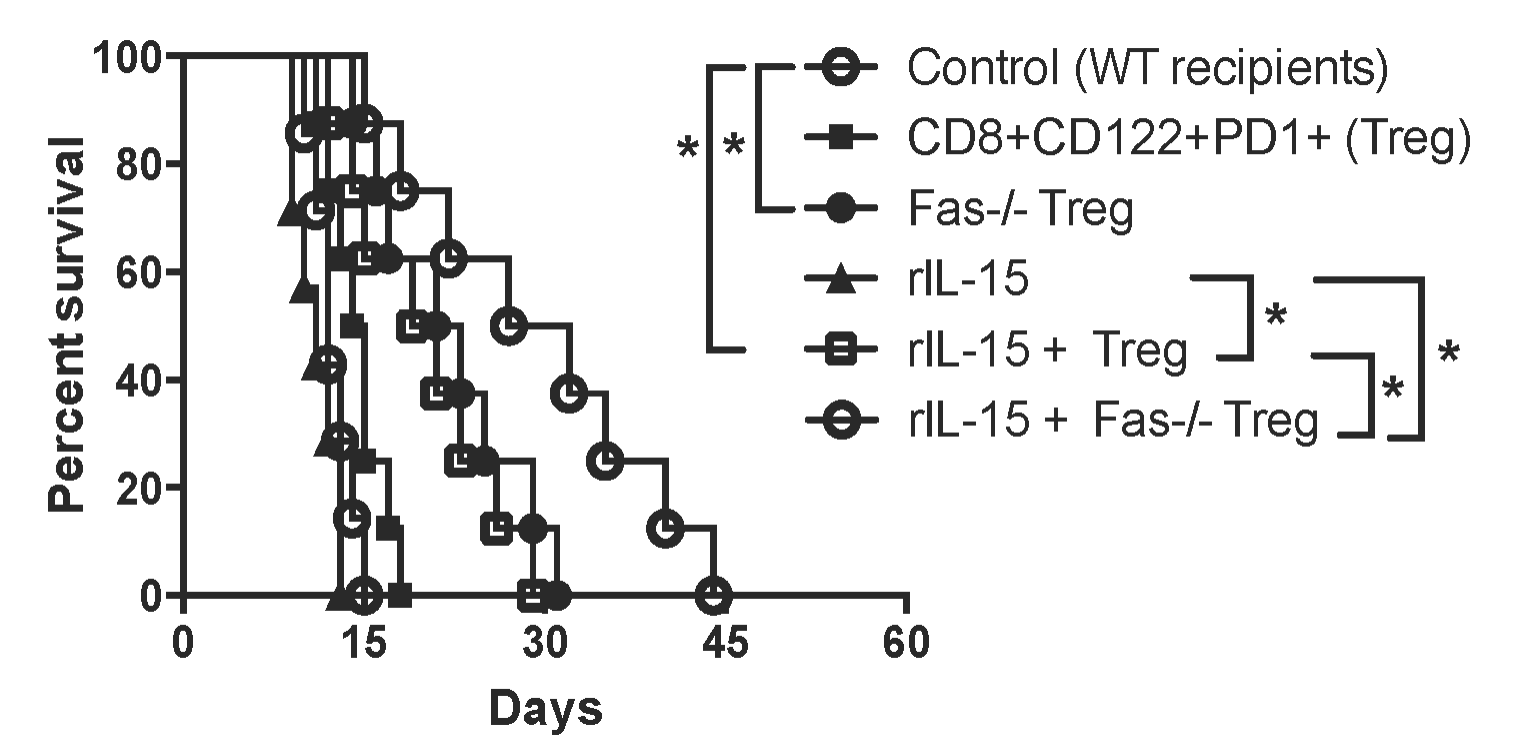
Fas-deficiency in CD8+CD122+PD-1+ Tregs or administration of rIL-15 enhances their suppression of skin allograft rejection in wild-type mice Wild-type B6 mice were transplanted with a Balb/C skin and received WT (*n* = 8) or Fas-deficient CD8+CD122+PD-1+ Tregs (*n* = 8). Some recipients were also treated with recombinant rIL-15 (*n*= 7) or both rIL-15 and the Tregs (*n* = 8). A lack of Fas receptor on the Tregs synergized with administration of IL-15 to further enhance their inhibition of skin allograft rejection in immune competent wild-type mice. (*P < 0.05, *n* = 7-8).

**Figure 5:**
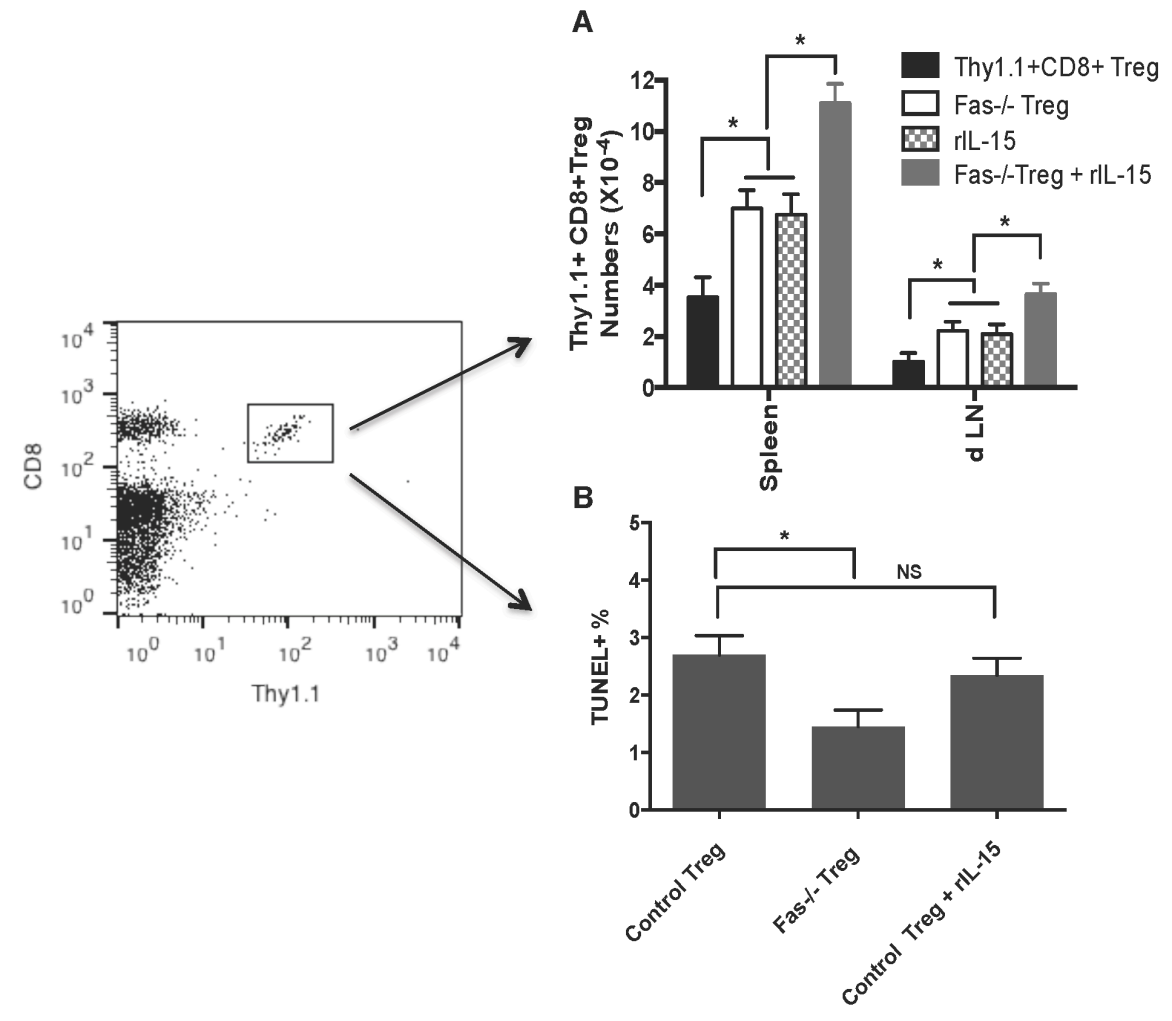
Fas-deficiency or administration of IL-15 expands CD8+CD122+PD-1+ Tregs Wild-type B6 mice were transplanted with Balb/C skin and received Fas-replete or Fas-deficient CD8+CD122+PD-1+ Tregs isolated from Thy1.1+ or Fas−/−Thy1.1+ mice. Some of the recipients were also treated with recombinant rIL-15. Ten days later, CD8+Thy1.1+ Tregs in spleens and draining LNs (dLN) of recipients were enumerated by flow analyses (**A**.) Similarly, dLN cells were analyzed for CD8+Thy1.1+ Treg cell apoptosis by TUNEL (**B**.) Data are presented as mean ± SD. One representative of three separate experiments is shown.

To be clinically relevant, Tregs need to be effective in suppression in immune competent wild-type animals. We asked whether or not lacking Fas death receptor would enhance CD8+CD122+PD1+ Treg suppressive function in wild-type recipients. We also examined if administering recombinant rIL-15 would increase their suppressive capacity given that IL-15 has been shown to be critical for the generation and maintenance of CD8+ memory (CD8+CD122+CD44^high^) T cells. Wild-type B6 mice were transplanted with Balb/C skin and received Fas-replete or Fas-deficient CD8+CD122+PD-1+ Tregs, and were treated with recombinant rIL-15. As shown in Figure 4, the adoptive transfer of the Tregs derived from Fas-deficient mice (MST= 22 vs. 12 days, n=7-8, P<0.05), but not wild-type mice (MST= 14 vs. 12 days, n=7-8, P>0.05), significantly delayed skin allograft rejection. Administration of rIL-15 alone also prolonged skin allograft survival (MST= 20 vs. 12 days, n=7-8, P<0.05). Importantly, the combined approaches with both Fas-deficient Treg transfer and administration of rIL-15 further extended the allograft survival (MST= 30 vs. 22 days, n=8, P<0.05). To determine if these measures enhanced the Treg suppression of allograft rejection by promoting their expansion *in vivo*, similarly transplanted wild-type recipients received Fas-replete or Fas-deficient CD8+CD122+PD-1+Thy1.1+ Tregs and/or rIL-15. As shown in Figure 5A, the numbers of Fas-deficient Thy1.1+ Tregs in both spleens and draining lymph nodes (dLN) of recipients were increased compared to those of Fas-replete Thy1.1+ Tregs 10 days following transplantation. Administration of rIL-15 also significantly augmented the Treg numbers while the combined measures with both transfer of Fas-deficient Tregs and administration of rIL-15 further increased their numbers. Similar findings were also observed 20 days after transplantation (data not shown). On the other hand, the Fas-deficient Thy1.1+ Tregs derived from dLNs of recipients were increasingly resistant to apoptosis compared with the control Tregs (Figure 5B) whereas administration of IL-15 did not alter their apoptotic rates. These data suggest that Fas-deficient CD8+CD122+PD-1+ Tregs undergo faster expansion than do the Fas-replete Tregs, especially in the presence of exogenous IL-15.

## DISCUSSION

Using skin allotransplant and adoptive T-cell transfer models of lymphocyte-deficient mice as well as wild-type recipients, we studied the mechanisms by which CD8+CD122+PD-1+ Tregs exert their suppression of alloimmune responses. We found that inhibition of skin allograft rejection by the Tregs was mostly dependent on their expression of Fas ligand. Their suppression was also largely reversed when effector T cells lacked Fas receptor. FasL+ Tregs induced conventional T cell apoptosis *in vitro* in a FasL-Fas-dependent manner. Moreover, Treg adoptive transfer significantly extended the allograft survival even in wild-type murine recipients if the CD8+ Tregs lacked Fas receptor or recipients received recombinant IL-15, because these two approaches synergistically expanded the Tregs transferred to wild-type recipients. Therefore, this study revealed a new mechanism underlying the CD8+ Treg suppression of allograft rejection, and could be implicated for Treg therapies in clinical transplantation.

The mechanisms underlying CD8+CD122+PD-1+ Treg suppression remain not well understood. IL-10 plays an important role in CD4+CD25+ Treg-mediated suppression [22–24]. IL-10 production by CD8+CD122+ Tregs has also been shown to be one of the mechanisms underlying their suppression [10, 18, 25]. CD8+CD122+ Tregs suppressed the proliferation of CD8+ T cells by producing IL-10 [10]. They also recognized conventional T cells through their interaction with MHC class I-αβ TCR and regulated T cell responses through IL-10 production [25]. Others demonstrated that CD8+CD122+ Tregs from RasGRP1(−/−) mice inhibited the proliferation of CD8+CD122- T cells also through IL-10 [26]. We previously found that suppression of allograft rejection by CD8+CD122+PD-1+ Tregs was partially dependent on IL-10 [18] and that PD-1 signaling was required for their maximal production of IL-10. Hence, other mechanisms may be also involved in CD8+CD122+PD-1+ Treg-mediated suppression of allograft rejection.

CD8+ cytotoxic T lymphocytes (CTL) exert their effector functions through two signaling pathways: Perforin/granzyme and FasL/Fas. Granzymes enter the target cell cytoplasm and their serine protease triggers the caspase cascade, leading to target cell apoptosis. Engagement of Fas with FasL initiates the recruitment of death-induced signaling complex (DISC). Then Fas-associated death domain (FADD) translocates with the DISC and recruits pro-caspases 8 and 10 that in turn activate the effector caspases 3 and 6 etc., eventually leading to the apoptosis of Fas+ target cells. Indeed, we found that suppression of alloimmune responses by CD8+CD122+PD-1+ Tregs was mostly dependent on their expression of FasL, but not perforin, and that the Tregs also induced conventional T cell apoptosis *in vitro* in a FasL/Fas-dependent manner. Previous studies demonstrated that CD4+ Treg cells restricted effector T cell generation through a Fas Ligand-dependent mechanism [27] and maintained allograft tolerance in a granzyme B-dependent manner [28], suggesting that both pathways may be involved in CD4+ Treg-mediated suppression. It remains to be defined why FasL/Fas, but not perforin/granzyme, pathway is involved in CD8+CD122+PD1+ Treg-mediated suppression of alloimmune responses. Perhaps, the suppression of alloimmune responses through FasL-Fas interactions in our experimental models simply results from the physical contacts between CD8+CD122+PD-1+ Tregs and Fas+ effector T cells.

Our data indicate that administration of rIL-15 at low doses enhances the suppression of allograft rejection in wild-type mice by CD8+CD122+PD1+ Tregs, which otherwise would have been ineffective in immune competent recipients. Their transfer alone did not work in immune competent recipients may be due to the insufficient numbers. It is also possible that some CD8+CD122+PD-1+ Tregs may lose their expression of PD-1 following adoptive transfer. However, the number of adoptively transferred Tregs was much higher after administering rIL-15 than that of control Tregs without IL-15, suggesting that IL-15 enhances the Treg suppression by expanding them *in vivo*, likely through promoting their homeostatic proliferation. Hence, our findings could have important implications for Treg cell therapies in clinical transplantation. Previous studies have shown that IL-15 is critical for the generation and maintenance of memory CD8+ T cells [29, 30] while administration of recombinant IL-15 increases their precursor frequency *in vivo* [31, 32]. Therefore, it is understandable that IL-15 increases the suppressive capacity of CD8+CD122+PD1+ Tregs by expanding these Tregs since they also exhibit a CD8+ memory phenotype. However, IL-15 could also promote the generation of endogenous and donor-specific memory CD8+ T cells that do more harm than good to an allograft. In our studies, we successfully used low doses of IL-15 to promote expansion of CD8+CD122+PD-1+ Tregs that significantly inhibited allograft rejection, indicating that administration of IL-15 does not significantly increase donor-specific memory CD8+ T cells, which would otherwise damage an allograft. Perhaps, adoptively transferred CD8+CD122+PD-1+ Tregs can easily outnumber endogenous, harmful and donor-specific CD8+ memory T cells since endogenous T cell memory is generally developed in a very small number.

## MATERIALS AND METHODS

### Mice and antibodies

Wild-type BALB/c (H-2^d^) and C57BL/6 (H-2^b^) mice were purchased from Guangdong Medical Laboratory Animal Center (Fushan, Guangdong, China) and National Cancer Institute (NIH, Bethesda, MD, USA). Rag1−/−, Fas−/− (lpr), FasL−/− (gld), Perforin−/− and Thy1.1+ congenic mice were all in B6 background and purchased from the Jackson Laboratory (Ann Arbor, MI). Fas−/− mice were also backcrossed to a Thy1.1+ background to establish Thy1.1+Fas−/− colony. All mice were housed in a specific pathogen-free (SPF) environment, and all animal experiments were approved by the institutional animal care and use committee (IACUC). Recombinant murine IL-15 mAb and neutralizing anti-IL-10 mAb were bought from eBioscience (San Diego, CA) while blocking anti-FasL mAb, activating anti-CD3 and anti-CD28 mAbs, and Abs for flow analysis, including anti-CD8-PE, anti-CD122-FITC, anti-PD-1-PerCP, anti-FasL-biotin and anti-Thy1.1-PerCP, were purchased from BD Biosciences (Mountain View, CA).

### Skin transplantation

Skin donors were 7-8-week-old wild-type BALB/c mice while skin allograft recipients were 7-8-week-old Rag1−/− or WT C57BL/6 mice. Full-thickness trunk skin was transplanted to the dorsal flank area of recipient mice and secured with the bondage of Band-Aid (Johnson Johnson, New Brunswick, NJ). Skin rejection was defined as graft necrosis greater than 90% as described in our previous publications [18, 33].

### Treatments of mice

Rag1−/− recipients received 1×10^6^ CD8+CD122+PD-1+ Tregs and/or 4×10^6^ CD3+ T cells one day following skin transplantation. In some wild-type recipients, recombinant murine IL-15 was administered at 2 μg/day on days 2, 4, 6, 8, 10 and 14 while blocking anti-FasL mAb was injected *i.p*. at 0.1 mg on days 2, 4, 6, 8, 10 and 14 following transplantation in either Rag1−/− or wild-type recipients. The latter, however, received larger numbers of CD8+CD122+PD-1+ Tregs (2×10^6^ per mouse).

### CD8+CD122+PD-1+ Treg isolation

Spleen cells from 6-7 week-old naïve B6 mice were pooled after lysing red blood cells. Cells were then stained with anti-CD8-PE, anti-CD122-FITC, anti-PD-1-PerCP and anti-CD3-APC mAbs (BD Biosciences, Mountain View, CA), and CD8+CD122+PD-1+ Tregs or CD3+ T cells were sorted out using a FACSAria III (BD Biosciences). The purity of the sorted cells was typically > 95%.

### Flow analysis

To determine if CD8+CD122+PD-1+ Tregs express FasL, cells were stained with anti-CD8-PE, anti-CD122-FITC, anti-PD-1-PerCP, and anti-FasL-biotin followed by streptavidin-APC. Cells were washed and analyzed using a FACSCalibur (BD Biosciences). To enumerate transferred CD8+CD122+PD-1+Thy1.1+ Tregs, they were stained with anti-CD8-PE and anti-Thy1.1-PerCP mAbs and washed twice before flow analysis using a FACSCalibur.

### Analysis of T cell proliferation *in vitro* for Treg suppression assays

CD8+CD122+PD-1+ Tregs from naive B6 mice were first isolated by FACS sorting. They were then cultured with B6-derived T cells (Teff), which were enriched via nylon wool columns (Polysciences, Warrington, PA), in 96-well plates in the complete RPMI 1640 medium (10%FCS, 2mM glutamine, 100U/ml penicillin, and 100μg/ml streptomycin). The ratios of Treg to Teff were 1:4 (Treg: 1×10^5^/well and Teff: 4×10^5^/well). Irradiated BALB/c spleen cells (2.5×10^5^/well) were added to the culture to serve as donor-derived stimulators, as described previously [33, 34]. Three and five days later, cells were harvested and analyzed by a Scintillation counter (PerkinElmer, Meriden, CT). Cells were pulsed with [^3^H]-Thymidine for the last 8 hours before analysis.

### Analysis of T cell apoptosis by a TUNEL method

To detect cell apoptosis *in vitro*, FACS-sorted CD8+CD122+PD-1+Thy1.1+ Tregs and CD3+ T cells were cultured in the presence of anti-CD3 and anti-CD28 Abs (2.5ng/ml) for 72 hours. For cell apoptosis *in vivo*, dLN cells were directly isolated from recipients. Cells were stained for surface markers Thy1.1 and CD8. They were then fixed in 2% paraformaldehyde, permeabilized with 0.1% Triton X-100 solution, and labeled with fluorescein-tagged deoxyuridine triphosphate (dUTP) by the terminal deoxynucleotidyl transferase-mediated dUTP nick-end labeling (TUNEL) method according to the manufacturer's instructions (Roche Applied Science, Mannheim, Germany).

### Statistical analysis

Comparisons of the mean were performed using ANOVA. The analysis of graft survival was conducted using Kaplan-Meier method (log-rank test). All analyses were performed using Prism-6 software (GraphPad Software, La Jolla, CA). Data were presented as Mean ± SD. A value of P<0.05 was considered statistically significant.

## Abbreviations

dLN: draining lymph node
FasL: Fas ligand
MST: median survival time
Treg: regulatory T cell
TUNEL: terminal deoxynucleotidyl transferase-mediated dUTP nick-end labeling
